# Efficacy of parainfluenza virus 5 (PIV5)-vectored intranasal COVID-19 vaccine as a single dose primer and booster against SARS-CoV-2 variants

**DOI:** 10.1128/jvi.01989-24

**Published:** 2025-03-21

**Authors:** Ashley C. Beavis, Zhuo Li, Kelsey Briggs, María Cristina Gingerich, Elizabeth R. Wrobel, Maria Najera, Dong An, Nichole Orr-Burks, Jackelyn Murray, Preetish Patil, Jiachen Huang, Jarrod Mousa, Linhui Hao, Tien-Ying Hsiang, Michael Gale, Stephen B. Harvey, S. Mark Tompkins, Robert Jeffrey Hogan, Eric R. Lafontaine, Hong Jin, Biao He

**Affiliations:** 1 CyanVac LLC, Athens, Georgia, USA; 2 Department of Infectious Diseases, College of Veterinary Medicine, University of Georgia 551782 , Athens, Georgia, USA; 3 Department of Immunology, Center for Innate Immunity and Immune Disease, University of Washington 198634 , Seattle, Washington, USA; 4 Animal Resources, University of Georgia 1355 , Athens, Georgia, USA; 5 Department of Population Health, College of Veterinary Medicine, University of Georgia 308501 , Athens, Georgia, USA; St. Jude Children's Research Hospital, Memphis, Tennessee, USA

**Keywords:** SARS-CoV-2, viral-vectored vaccine, parainfluenza virus 5 (PIV5), golden Syrian hamster

## Abstract

Immunization with COVID-19 vaccines has greatly reduced COVID-19-related deaths and hospitalizations from SARS-CoV-2 infection, but waning immunity and the emergence of variants capable of immune escape indicate the need for annual vaccine updates or development of different SARS-CoV-2 vaccine platforms. Parainfluenza virus 5 (PIV5)-vectored intranasal COVID-19 vaccine with the ancestral spike (S) protein (CVXGA1) has been shown to be a promising next-generation COVID-19 vaccine preclinically and is currently being evaluated in humans. This work investigates the immunogenicity and efficacy of CVXGA1 and other PIV5-vectored vaccine candidates expressing additional SARS-CoV-2 nucleoprotein (N) antigen or SARS-CoV-2 variant S proteins of beta, delta, gamma, and omicron variants against homologous and heterologous challenges in hamsters. A single intranasal dose of CVXGA1 induces neutralizing antibodies (nAbs) against SARS-CoV-2 WA1 (ancestral), delta variant, and omicron variant and protects against both homologous and heterologous virus challenges. Compared to mRNA COVID-19 vaccine, neutralizing antibody titers induced by CVXGA1 were well maintained over time. When administered as a booster following two doses of an mRNA COVID-19 vaccine, PIV5-vectored vaccines expressing the S protein from WA1 (CVXGA1), delta, or omicron variants generate higher levels of cross-reactive nAbs than three doses of mRNA vaccine. Our data indicate that an intranasal PIV5-vectored COVID-19 vaccine can serve as a booster vaccine against emerging variants.

**IMPORTANCE:**

With emerging new variants of concern (VOC), SARS-CoV-2 continues to be a major threat to human health. Approved COVID-19 vaccines have been less effective against these emerging VOCs. This work demonstrates the protective efficacy and strong boosting effect of an intranasal viral-vectored vaccine against SARS-CoV-2 variants in hamsters. Our intranasal vaccine can act as an effective booster for individuals already 58 vaccinated against SARS-CoV-2.

## INTRODUCTION

SARS-CoV-2 first emerged in Wuhan, China in December 2019 (1). Since then, numerous SARS-CoV-2 variants have emerged. The World Health Organization defines a SARS-CoV-2 variant of concern (VOC) as a variant that affects virus transmissibility and COVID-19 epidemiology, increases virulence and pathogenicity, or decreases the effectiveness of COVID-19 vaccines (immune escape). The current VOC is of omicron lineage, while previously circulating VOCs include alpha, beta, gamma, and delta (https://www.who.int/activities/tracking-SARS-CoV-2-variants).

Currently, four SARS-CoV-2 vaccines are approved for use in the United States (2, 3). As of July 2023, over 13 billion vaccine doses have been administered worldwide (https://covid19.who.int) with approximately 17% of the United States population having received a bivalent booster vaccination (4). However, SARS-CoV-2 variants have demonstrated immune escape in previously infected and fully vaccinated individuals. Sera from individuals who received two doses of Pfizer’s mRNA vaccine had neutralizing antibody titers against delta variant three- to fivefold lower than alpha variant (5). A study found that the omicron-neutralizing ability of serum from WA1-convalescent individuals was eightfold lower than its WA1-neutralizing ability (6), and recently circulating omicron subvariant XBB.1 was found to greatly evade neutralizing antibodies (nAbs) of individuals who received the BA.5 bivalent mRNA booster vaccine (7, 8). For vaccinated individuals, vaccine efficacy significantly decreased within 1–6 months after full vaccination, which was associated with waning immunity (9). Waning immunity and the emergence of variants capable of immune escape indicate an urgent need for developing additional SARS-CoV-2 vaccine candidates with long-lasting protective immunity against the variants.

Parainfluenza virus 5 (PIV5) is a negative-sense, single-stranded, RNA virus in the family *Paramyxoviridae* (10, 11). Because it actively replicates in the respiratory tract following intranasal (IN) immunization, PIV5-vectored vaccines can generate antigen-specific cellular responses and mucosal immunity that includes antigen-specific IgA antibodies and long-lived IgA plasma cells (12, 13). A PIV5-vectored vaccine expressing the spike protein from SARS-CoV-2 Wuhan (ancestral strain; WA1; CVXGA1) has been shown to be efficacious in mice and ferrets (14). A single, intranasal dose of CVXGA1 induced WA1-nAbs and protected K18-hACE2 mice against lethal infection with SARS-CoV-2 WA1. Furthermore, a single, intranasal dose of CVXGA1 protected ferrets from SARS-CoV-2 WA1 infection and blocked contact transmission to cohoused naïve ferrets (14). This intranasal vaccine, CVXGA1, has completed phase 1 clinical study (15) and is currently under phase 2 clinical evaluation in the US (16).

While previous studies demonstrated its efficacy against SARS-CoV-2 WA1, CVXGA1’s efficacy against SARS-CoV-2 variants was not tested. To address this, a golden Syrian hamster model was utilized. Golden Syrian hamsters have been proven susceptible to infection with SARS-CoV-2. Following infection with WA1, hamsters lose weight for 6 days before starting to recover, and SARS-CoV-2 viral RNA can be detected in nasal turbinate, trachea, and lungs of infected hamsters with peak infectious viral titers measured at 4 days post-infection (17). Several studies have shown that hamsters are also susceptible to infection with alpha and delta variants (18–20). Among VOC alpha, delta, and omicron, delta variant causes the most weight loss and replicates at the highest level in the lungs of infected hamsters. In contrast, omicron VOC, even with a high-dose infection (2.5 × 10^6^ PFU per animal) did not cause weight loss and replicates poorly in the lower respiratory tract of infected hamsters (21).

In this work, we examined the efficacy of CVXGA1 and other recombinant PIV5 vaccines expressing S from SARS-CoV-2 beta, gamma, delta, or omicron, as a single-dose intranasal vaccine and as a booster following vaccination with two doses of COVID-19 mRNA vaccine against challenge infection with WA1, alpha, and delta in a golden Syrian hamster model. We also evaluated a vaccine virus expressing both the S and N protein from SARS-CoV-2 WA1 strain in hamsters.

## MATERIALS AND METHODS

### Cells

Vero E6 cells were maintained in Dulbecco’s modified Eagle medium (DMEM) supplemented with 5% fetal bovine serum (FBS) and 100 IU/mL penicillin/100 µg/mL streptomycin (1% P/S; Mediatech Inc, Manassas, VA, USA). Serum-free (SF) Vero cells were maintained in VP-SFM (ThermoFisher Scientific) plus 4 mM GlutaMax (Gibco). Vero-TMPRSS2 cells were obtained from Dr. Jeff Hogan, University of Georgia, and maintained in DMEM + 10% FBS + 1 mg/mL G418. All cells were incubated at 37°C, 5% CO_2_. Cells are frequently tested for mycoplasma. The cell line used to generate the vaccines was validated via karyology testing.

### Plasmids and virus rescue

The construction of a plasmid encoding for PIV5 antigenome and generation of recombinant PIV5 were as previously described (22). To construct plasmids encoding the antigenome of CVXGA1, CVXGA3, CVXGA5, CVXGA13, and CVXGA14, the Spike (S) genes from SARS-CoV-2 WA1, alpha, gamma, delta, and omicron, respectively, were placed as an additional open reading frame (ORF) transcription unit between the PIV5 SH and HN genes. The S cytoplasmic tail was replaced by the PIV5 fusion (F) protein cytoplasmic tail. To construct CVXGA2, encoding both the SARS-CoV-2 WA1 nucleoprotein (N) and S proteins, the N gene was placed as an additional ORF transcription unit between the PIV5 HN and L genes of CVXGA1. Primer sequences are available upon request. To generate recombinant PIV5 viruses CVXGA1, CVXGA2, CVXGA3, CVXGA5, CVXGA13, and CVXGA14, plasmids encoding the PIV5 antigenomic cDNA, the supporting plasmids (PIV5-NP, P, and L), and T7 polymerase were transfected into SF Vero cells by FuGene transfection reagent (Fugent) or electroporation (Neon transfection system, Invitrogen). The recovered virus was amplified in SF Vero cells, and the viral genomes were verified by RT-PCR and Sanger sequencing.

### Virus propagation

The recombinant PIV5 viruses were propagated in SF Vero cells at a multiplicity of infection (MOI) 0.001 PFU in VP-SFM + 4 mM GlutaMax for 5–7 days at 37°C with 5% CO_2_. The media was collected and centrifuged at 1,500 rpm for 10 min to pellet cell debris. The supernatant was mixed with 0.1 vol of 10× sucrose-phosphate-glutamate (SPG) buffer or 10× SPG + 10% Arginine, aliquoted, flash-frozen in liquid nitrogen, and stored at −80°C. The PIV5 virus stocks were titrated by plaque assay in Vero cells followed by immunostaining.

The SARS-CoV-2 viruses were propagated in Vero cells with DMEM + 1% FBS + 1× P/S. WA1 (BEI NR-52281) and alpha variant (USA/CA_CDC_5574/2020; BEI NR-54011) were obtained from BEI Resources. The omicron BA1 variant was provided by Dr. Jeff Hogan, University of Georgia. The delta variant was provided by Dr. Michael Gale, Jr., University of Washington. For isolation and production of delta variant stock, SARS-CoV-2 positive specimens with cycle threshold (CT) <33 were identified from reference testing (23). The positive specimens were transferred to a biosafety level (BSL) 3 laboratory for virus culture. The virus transport medium (VTM) was first cleaned by filtering through Corning Costar Spin-X centrifuge tube filter (CLS8160), and 0.1 mL of the cleaned VTM was used to infect Vero E6 cells ectopically expressing human ACE2 and TMPRSS2 (VeroE6-AT cells; a gift from Dr. Barney Graham, National Institutes of Health, Bethesda MD) in a 48-well plate. Two to four days post-infection when cytopathic effect, typical of SARS-CoV-2 infection, was observed, and culture supernatants were collected and designated as a passage P0 virus stock. P1 virus stock cultures were grown in Vero E6/TMPRSS2 cells (JCRB1819) using P0 virus as inoculum. The titer of the P1 stock was measured by standard SARS-CoV-2 plaque assay as described (24). P1 stock virus was verified with whole-genome sequencing on an Illumina NextSeq 500 (Illumina, San Diego, CA, USA) along with positive and negative controls. The delta variant stock is defined as Pangolin B.1.617.2.

### Immunofluorescence assay

Immunofluorescence assays were performed to examine protein expression in virus-infected Vero cells. Vero cells were infected at an MOI of 0.01 with PIV5, CVXGA1, CVXGA2, CVXGA3, CVXGA5, CVXGA13, or CVXGA14 for 3 days before being fixed with 80% methanol. The cells were incubated with mouse PIV5-specific V/P monoclonal antibody (PK 366) that was previously described (25), rabbit anti-SARS-CoV-2 S (Sino Biological catalog no. 40150-R007), or SARS-CoV-2 N (ProSci catalog no. 35–579) antibodies at 1:500 in phosphate buffered saline (PBS) + 3% bovine serum albumin (BSA) for 1 h. Next, the cells were washed with PBS and incubated with goat α-mouse Cy3 (KPL) or goat α-rabbit Cy3 (KPL) at 1:500 in PBS + 3% BSA for 30 min. The cells were washed with PBS and imaged with an EVOS M5000 microscope (Thermo Fisher Scientific).

### Hamsters

Five- to 7-week-old Golden Syrian hamsters were obtained from Charles River Laboratories. The hamsters were single housed in animal BSL2 (ABSL2) facilities with *ad libitum* access to food and water. Pre-challenge procedures were performed at the University of Georgia Biological Sciences Animal Facility. The hamsters were transferred to BSL3 facilities in the University of Georgia Animal Health Research Center (ABSL3) for the challenge and post-challenge procedures. The hamsters were anesthetized for immunization, blood collection, and challenge by intraperitoneal injection of 100 µL ketamine/acepromazine cocktail.

Hamsters were assigned to vaccine groups randomly. There was no blinding of vaccine groups during the experiments.

### Immunization and challenge of hamsters

To administer intranasal immunizations, anesthetized hamsters were placed on their backs, a pipette was used to dispense 100 µL inoculum onto their noses, and the inoculum was allowed to drain into their respiratory tracts. They were recovered on heating pads.

A COVID-19 mRNA vaccine was obtained from a clinical site, reconstituted to 200 µg/mL, aliquoted, and stored at −80°C. Two micrograms of mRNA vaccine in 50 µL was administered via intramuscular (IM) injection.

For study AE19 (Fig. 3 and 4), hamsters (*n* = 8) received a single intranasal immunization of 100 µL of 10^5^ PFU PIV5, CVXGA1, CVXGA2, CVXGA3, or CVXGA5. At 28 days post-immunization (dpi), blood was collected from the hamster saphenous vein. At 36 dpi, four hamsters were challenged intranasally with 30 µL 10^3^ PFU of SARS-CoV-2 Wuhan strain (WA1), and the remaining four hamsters were challenged with 10^3^ PFU SARS-CoV-2 alpha variant (CA; BEI NR54011) as previously reported by Blanchard, et al. (26). Following challenge infection, the hamster weights were monitored for 5 days. At 5 days post-challenge (dpc), the hamsters were euthanized, and the hamster lungs were harvested, resuspended in 2 mL DMEM + 2% FBS + 1× antibiotic/antimycotic, homogenized, aliquoted, and stored at −80°C. SARS-CoV-2 viral burden in lung homogenate was quantified via plaque assay and real-time quantitative reverse transcription PCR (RT-qPCR).

For study AE23 (Fig. 5), hamsters received one immunization (*n* = 5). Hamsters were intranasally immunized with 100 µL of PBS (group 1), 3 × 10^5^ PFU CVXGA1 (group 2), 2 × 10^5^ PFU CVXGA3 (group 3), or 1.5 × 10^5^ PFU CVXGA13 (group 4). Blood was collected from the hamster saphenous veins at 19 dpi. At 28 dpi, the hamsters were challenged with 10^4^ PFU SARS-CoV-2 delta variant. Following challenge infection, hamster weights were monitored for 5 days. At 5 dpc, the hamsters were euthanized, their lungs were harvested, and the SARS-CoV-2 viral burden was quantified via plaque assay and RT-qPCR. Hamsters were anesthetized for immunizations, blood collection, and challenge.

For study AE24 (Fig. 6 and 7), hamsters were immunized three times. For the first immunization, hamsters received 100 µL of PBS IN (*n* = 5, group 1 “PBS”), 2 µg mRNA COVID-19 vaccine IM(*n* = 25, group 2), or 100 µL 7 × 10^4^ PFU CVXGA1 IN (*n* = 10, group 3 “2× CVXGA1” and group 4 “1× CVXGA1”). For the second immunization at 29 dpi, the hamsters that received the mRNA vaccine (group 2) were boosted with the mRNA vaccine IM and the group three hamsters received another dose of CVXGA1 IN (“2× CVXGA1”). For the third immunization at 91 dpi, hamsters who received two doses of mRNA received either 2 µg mRNA vaccine IM (*n* = 5, group 2A “3× mRNA”) or 100 µL of 7 × 10^4^ PFU CVXGA1 (*n* = 5, group 2B “CVXGA1”), 10^3^ PFU CVXGA13 (*n* = 5, group 2C “CVXGA13”), PBS IN (*n* = 5, group 2D “2× mRNA”), or 10^4^ PFU CVXGA14 (*n* = 5, group 2E “CVXGA14”). Hamsters were anesthetized for intranasal immunizations but not intramuscular injections. At 36 and 108 dpi, blood was collected via the hamster gingival vein. At 116 dpi, the hamsters were challenged with 30 µL of 10^5^ PFU SARS-CoV-2 delta variant. Following challenge infection, hamster weights were monitored for 5 days. The hamster lungs were harvested, and SARS-CoV-2 viral burden was quantified via plaque assay and RT-qPCR. Hamsters were anesthetized for blood collection and challenge.

### Enzyme-linked immunosorbent assay

To quantify the anti-SARS-CoV-2 S and receptor binding domain (RBD) humoral response, hamster serum was analyzed via enzyme-linked immunosorbent assay (ELISA). Immulon 2HB 96-well microtiter plates were coated with 100 µL SARS-CoV-2 S or RBD at 1 µg/mL. For all ELISAs, plates were coated with SARS-CoV-2 S and RBD from the WA1 strain, which were produced and purified as described previously (14). Hamster serum was serially diluted twofold and incubated on the plates for 2 h, followed by horseradish peroxidase-labeled goat anti-mouse IgG secondary antibody (Southern Biotech, Birmingham, Alabama) diluted 1:2,000 for 1 h. The plates were developed with KPL SureBlue Reserve TMB Microwell Peroxidase Substrate (SeraCare Life Sciences, Inc., Milford, Massachusetts), and OD_450_ values were obtained with a BioTek Epoch Microplate Spectrophotometer (BioTek, Winooski, Vermont). Antibody titers were calculated as log_10_ of the highest serum dilution at which the OD_450_ was greater than two SDs above the mean OD_450_ of naïve serum.

### Neutralization assays

To quantify the SARS-CoV-2 nAbs generated by the hamsters, microneutralization assays were performed in a BSL3 facility. Hamster serum was heat inactivated at 56°C for 45 min and serially diluted twofold. The serum was mixed 1:1 with 6 × 10^3^ focus-forming units (FFU)/mL SARS-CoV-2 WA1, delta, or omicron variants. The serum/virus mixture was incubated at 37°C for 1 h before being incubated on 96 wells of Vero cells for WA1 or Vero TMPRSS2 cells for delta and omicron, respectively. One hour post-infection, a methylcellulose overlay (DMEM + 5% FBS + 1% P/S + 1% methylcellulose) was added on top of the serum/virus mixture. The plates were incubated at 37°C, 5% CO_2_ for 24 h, fixed with 60% methanol/40% acetone, followed by immunostaining with anti-SARS-CoV-2 N antibody (ProSci catalog no. 35–579). The number of infected cells was quantified via Cytation 7 imaging reader (BioTek). Neutralization titers were calculated as log_10_ of the highest serum dilution at which the virus infectivity was reduced by at least 50%.

### Plaque assay for infectious virus titer

SARS-CoV-2 viral titer in lung homogenates was quantified by plaque assay using lung homogenates serially diluted in DMEM + 2% FBS + 1% antibiotic/antimycotic in Vero E6 cells for SARS-CoV-2 WA1 and alpha variant or Vero TMPRSS2 cells for delta and omicron variants in 12-well plates. At 1 h post-infection, the inoculum was removed, and a methylcellulose overlay (500 mL Opti-MEM + 0.8% methylcellulose + 2% FBS + 1% antibiotic/antimycotic) was added. Following incubation for 3 days, the cells were fixed with 60% methanol/40% acetone and stained with crystal violet, the number of plaques was counted, and viral titers were expressed as plaque-forming units per milliliter of lung homogenate.

### Quantitative PCR

SARS-CoV-2 viral RNA levels were quantified by RT-qPCR. SARS-CoV-2 virus was inactivated by mixing 100 µL lung homogenate with 900 µL TRIzol (Invitrogen). Using a QIAgen RNA extraction kit, RNA was extracted from 140 µL homogenate/TRIzol and eluted in 15 µL of elution buffer, of which 5 µL was used in the qRT-PCR. qRT-PCR was performed according to the protocol described in the “CDC 2019-Novel Coronavirus (2019-nCoV) Real-Time RT-PCR Diagnostic Panel…Instructions for Use” (page 26; https://www.fda.gov/media/134922/download) with Applied Biosystems TaqPath One Step RT qPCR Master Mix and SARS-CoV-2 Research Use Only qPCR Primer and Probe Kit primer/probe mix N1. To generate a standard curve, the viral titer was plotted on the x-axis, and the CT value was plotted on the y-axis. The standard curves were used to calculate the CT value that corresponds to 1 PFU per reaction (PFU/rxn) in virus stock and hamster lung homogenates. The CT value of RNA extracted from sterile elution buffer was designated the PCR negative cutoff.

### Statistics

All statistics were calculated using Prism version 9.3.1 (GraphPad Software, LLC.). For ELISA, neutralizing antibody, and RT-qPCR experiments (Fig. 3B, 4E and F, 5B, C, and F, 6B, C, and F, and 7A, B, and E), statistical significance was calculated by comparing vaccine groups to vector control, PBS, and/or mRNA boost groups by nonparametric Kruskal-Wallis multiple comparisons. For body weight graphs, statistical significance was calculated for each timepoint by comparing each vaccine group to the PBS group with Mann-Whitney tests (Fig. 4A and B, 5D, 6D, and 7C). The number of animals used was the minimum necessary to achieve an adequate sample size for the various experimental variables based on prior experience. All hamsters were randomly assigned to vaccine groups. Vaccination was not blinded, and no data were excluded from the analysis.

## RESULTS

### Construction and characterization of PIV5-vectored SARS-CoV-2 vaccines

**Fig 1:**
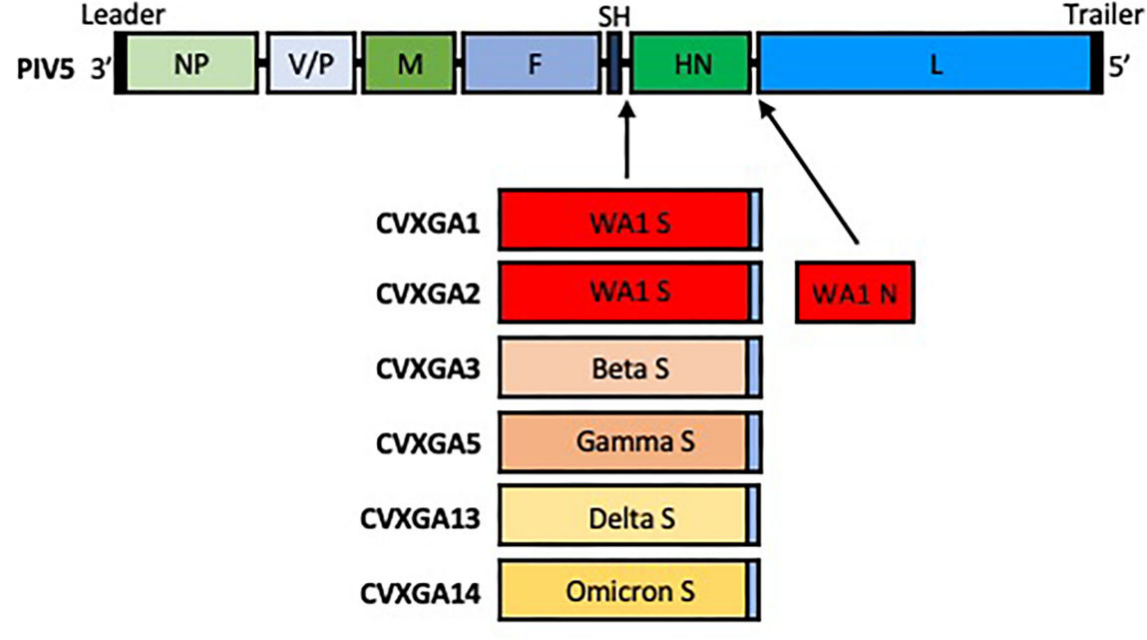
Schematics of PIV5 and CVXGA vaccines. The PIV5 genome has seven genes 3′ to 5′: nucleocapsid (NP) protein, V protein/phosphoprotein (V/P), matrix (**M**) protein, fusion (**F**) protein, small hydrophobic (SH) protein, hemagglutinin (HN), and polymerase (**L**). The spike (**S**) genes from SARS-CoV-2 WA1 (CVXGA1), beta variant (CVXGA3), gamma variant (CVXGA5), delta variant (CVXGA13), and omicron variant (CVXGA14) had their cytoplasmic tails replaced with the PIV5 F cytoplasmic tail and inserted between PIV5 SH and HN genes. CVXGA2 also has SARS-CoV-2 WA1 N inserted between PIV5 HN and L genes.

We previously generated a PIV5-vectored vaccine for SARS-CoV-2 by inserting the SARS-CoV-2 WA1 S gene, which had the cytoplasmic tail of the S protein replaced with the cytoplasmic tail from the PIV5 F protein, between the PIV5 SH and HN genes (CVXGA1). We showed that a single, intranasal dose of CVXGA1 protects K18-hACE2 mice from lethal infection with the WA1 strain, the initial circulating strain in the US, and blocks contact transmission in ferrets (14). To determine whether expressing the N protein of SARS-CoV-2 as an additional antigen enhances the protection afforded by the S antigen alone, we generated PIV5 expressing both S and N (CVXGA2). During the study period, SARS-CoV-2 VOCs emerged, and some became dominant strains at different times. Thus, we generated PIV5-vectored vaccine candidates expressing S from SARS-CoV-2 VOC in a similar manner as CVXGA1 (Fig. 1): variants beta (CVXGA3), gamma (CVXGA5), delta (CVXGA13), and omicron (CVXGA14; collectively called CVXGA vaccines).

**Fig 2:**
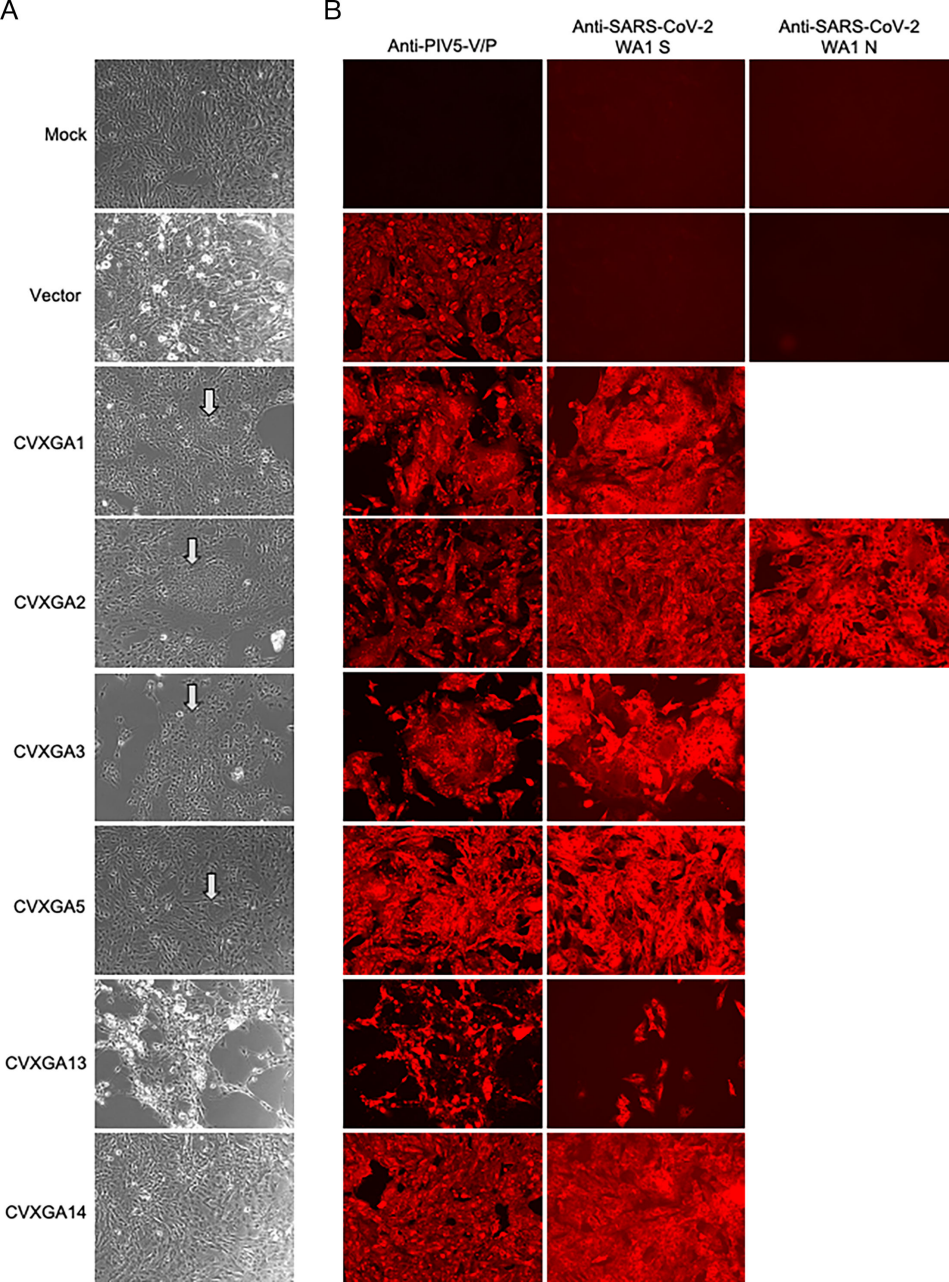
CVXGA vaccine antigen expression. Vero cells were infected at MOI 0.01 for 3 days. (**A**) Cell-to-cell fusion induced by SARS-CoV-2 S expression was imaged at 10× with an Evos M5000 microscope. Arrows indicate syncytium, multinucleated cells. (**B**) Intracellular expression of PIV5-V/P, SARS-CoV-2-S, and SARS-CoV-2-N was detected by anti-PIV5 V/P, -SARS-CoV-2 S, or SARS-CoV-2 N antibodies, followed by Cy3-conjugated secondary antibody, and imaged at 10× with an EVOS M5000 microscope (Thermo Fisher Scientific).

The inserted S and/or N antigen expression was confirmed by immunofluorescence assay in Vero cells infected with CVXGA1, 2, 3, 5, 13, or 14 using WA1 S-specific antibody or N-specific antibody for CVXGA2. SARS-CoV-2 N expression was detected in cells infected with CVXGA2 (Fig. 2B).

### PIV5-vectored SARS-CoV-2 vaccines induce anti-S humoral responses in hamsters

**Fig 3:**
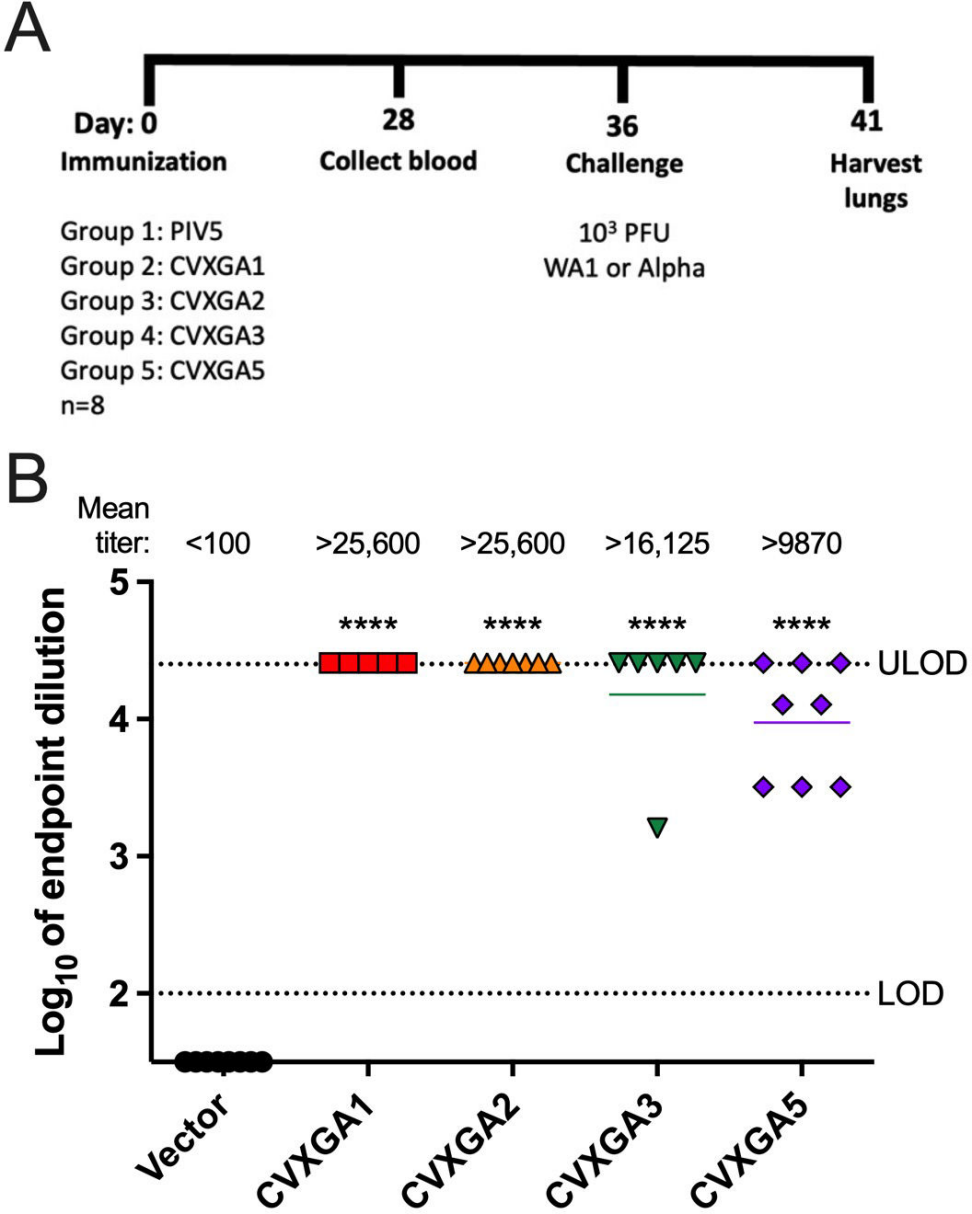
Immunization of hamsters with CVXGA1, CVXGA2, CVXGA3, and CVXGA5 induces anti-SARS-CoV-2 S IgG antibodies. (**A**) Schematic of hamster study AE19 immunization. Golden Syrian hamsters (*n* = 8) were intranasally immunized with 100 µL of 10^5^ PFU PIV5, CVXGA1, CVXGA2, CVXGA3, or CVXGA5. Blood was collected at 28 dpi. At 36 dpi, four hamsters from each group were challenged with 10^3^ PFU of SARS-CoV-2 Wuhan strain (WA1), and the remaining four hamsters were challenged with 10^3^ PFU SARS-CoV-2 alpha variant. Following challenge infection, the hamster weights were monitored daily before terminating the study at 5 dpc to collect lung tissues. (**B**) Anti-SARS-CoV-2 S IgG antibody titers were quantified by ELISA. Antibody titers were calculated as log_10_ of the highest serum dilution at which the OD_450_ was greater than two SDs above the mean OD_450_ of naïve serum. The lower limit of detection (LOD) and upper limit of detection (ULOD) are indicated by the dotted lines. Bars represent the geometric means. Comparing each group to the vector control, statistical significance was calculated by nonparametric Kruskal-Wallis multiple comparisons (*P ≤ 0.05, **P < 0.01, ***P < 0.001, and ****P < 0.0001).

The ability of PIV5-vectored COVID-19 vaccine viruses to induce S-specific antibody responses in hamsters was examined by immunizing golden Syrian hamsters with a single, intranasal dose of 10^5^ PFU of PIV5 vector, CVXGA1, CVXGA2, CVXGA3, or 5 × 10^2^ PFU CVXGA5 (Fig. 3A). While hamsters immunized with PIV5 vector had no detectable anti-SARS-CoV-2-S-binding antibodies at day 28 dpi, a single intranasal dose of CVXGA1, CVXGA2, or CVXGA3 induced mean ELISA antibody titers of greater than 16,125. CVXGA5, at a lower immunization dose, was able to induce an anti-S ELISA titer greater than 9,870 (Fig. 3B).

### CVXGA vaccines protect against homologous and heterologous challenges

**Fig 4:**
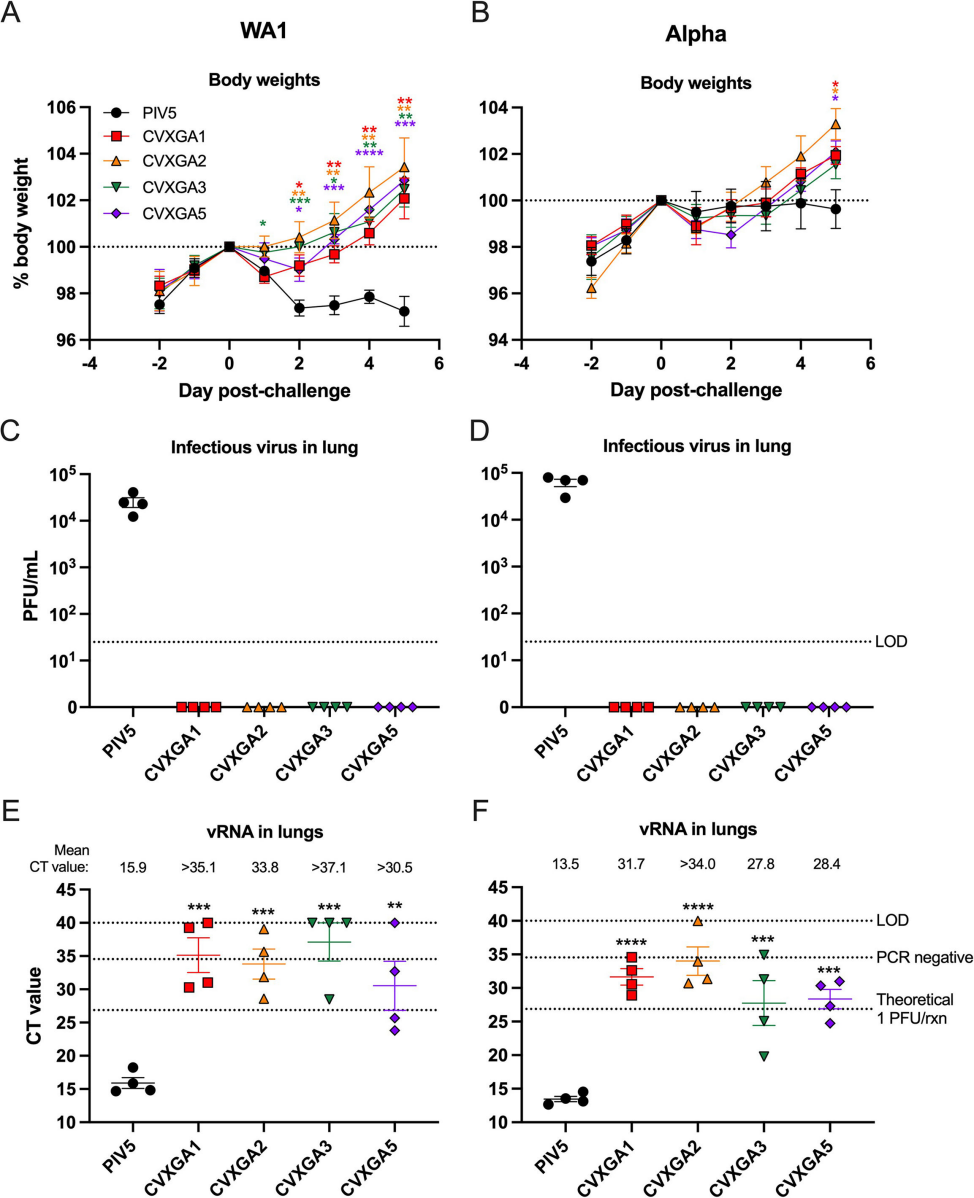
Immunization with CVXGA1 protects hamsters from challenge with SARS-CoV-2 WA1 and alpha variant. Following challenge with WA1 (**A**) or alpha variant (**B**), hamster weights were monitored daily for 5 days and graphed as percent day 0 wt. Statistics were calculated for each timepoint by comparing each vaccine group to the PBS group with Mann-Whitney tests (*P ≤ 0.05). At 5 dpc with WA1 (**C**) or alpha variant (**D**), viral load in hamster lung was quantified via plaque assay in Vero E6 cells and graphed as PFU/ml lung homogenate. The limit of detection (LOD) is indicated by the dotted line. Error bars represent the SEMs. SARS-CoV-2 WA1 (**E**) or alpha variant (**F**) vRNA load in lung homogenates was quantified via RT-qPCR. The CT value for each sample is presented, and error bars represent the SEMs. The known viral titers of WA1 and alpha variant were used to generate standard curves for E and F, respectively, and the CT values equating to 1 PFU/rxn were calculated. The CT value generated from RNA extracted from sterile water is denoted by a dotted line labeled PCR negative. The LOD is indicated by a dotted line at CT value = 40, the number of PCR cycles. Comparing each group to PIV5-immunized hamsters, statistical significance was calculated by nonparametric Kruskal-Wallis multiple comparisons (*P ≤ 0.05 and **P < 0.01).

To assess the efficacy of PIV5-vectored SARS-CoV-2 vaccines against homologous and heterologous virus challenges, CVXGA-immunized hamsters were challenged with either 10^3^ PFU SARS-CoV-2 WA1 (USA-WA01/2020) or alpha variant (CA; BEI NR54011) at 36 dpi. The hamster weights were monitored daily for 5 dpc. Following challenge with WA1, hamsters immunized with PIV5 vector lost weight and did not recover before the study was terminated. In contrast, hamsters immunized with CVXGA1, CVXGA2, CVXGA3, or CVXGA5 lost weight on day 1 post-challenge but returned to pre-challenge weights 3–4 dpc (Fig. 4A). While challenge with alpha variant induced less severe weight loss in PIV5 vector-immunized hamsters, hamsters immunized with CVXGA1, CVXGA2, CVXGA3, or CVXGA5 had significantly higher body weights compared to PIV5 vector-immunized hamsters at 5 dpc. In both virus challenge groups, hamsters immunized with CVXGA2 had the highest mean body weight gains following challenge (Fig. 4B), indicating that the SARS-CoV-2 N antigen provided additional protection.

The protection efficacy of vaccine viruses against SARS-CoV-2 challenge infection was examined by viral load in the lungs of hamsters. Animals immunized with PIV5 vector had infectious SARS-CoV-2 virus titer greater than 4 log_10_ PFU/mL lung homogenate following challenge with WA1 or alpha variant. No infectious WA1 or alpha variant was detected in hamsters immunized with CVXGA1, CVXGA2, CVXGA3, or CVXGA5 (Fig. 4C and D; limit of detection, LOD = 25 PFU/mL). Viral load in the lung tissues was also examined by a more sensitive RT-qPCR method to quantify challenge virus RNA levels. Hamsters immunized with PIV5 vector and challenged with WA1 or alpha variant had mean CT values of 15.9 and 13.5, respectively (Fig. 4E and F). Following challenge with WA1 or alpha variant, hamsters that received a single intranasal dose of CVXGA1 or CVXGA2 had CT values indicative of less than 1 PFU/rxn. CVXGA3-immunized hamsters had CT values indicative of less than 1 PFU/rxn following challenge with WA1 but two hamsters had CT values equating to 1 or 96 PFU/rxn following challenge with alpha variant (Fig. 4E and F; Fig. S1). A single dose of CVXGA2 performed the best against heterologous challenge with alpha variant (Fig. 4F) with significant viral load reduction compared to the control group (*P* < 0.01), suggesting that SARS-CoV-2 N might offer additional protection.

### CVXGA vaccines protect against delta challenge

**Fig 5:**
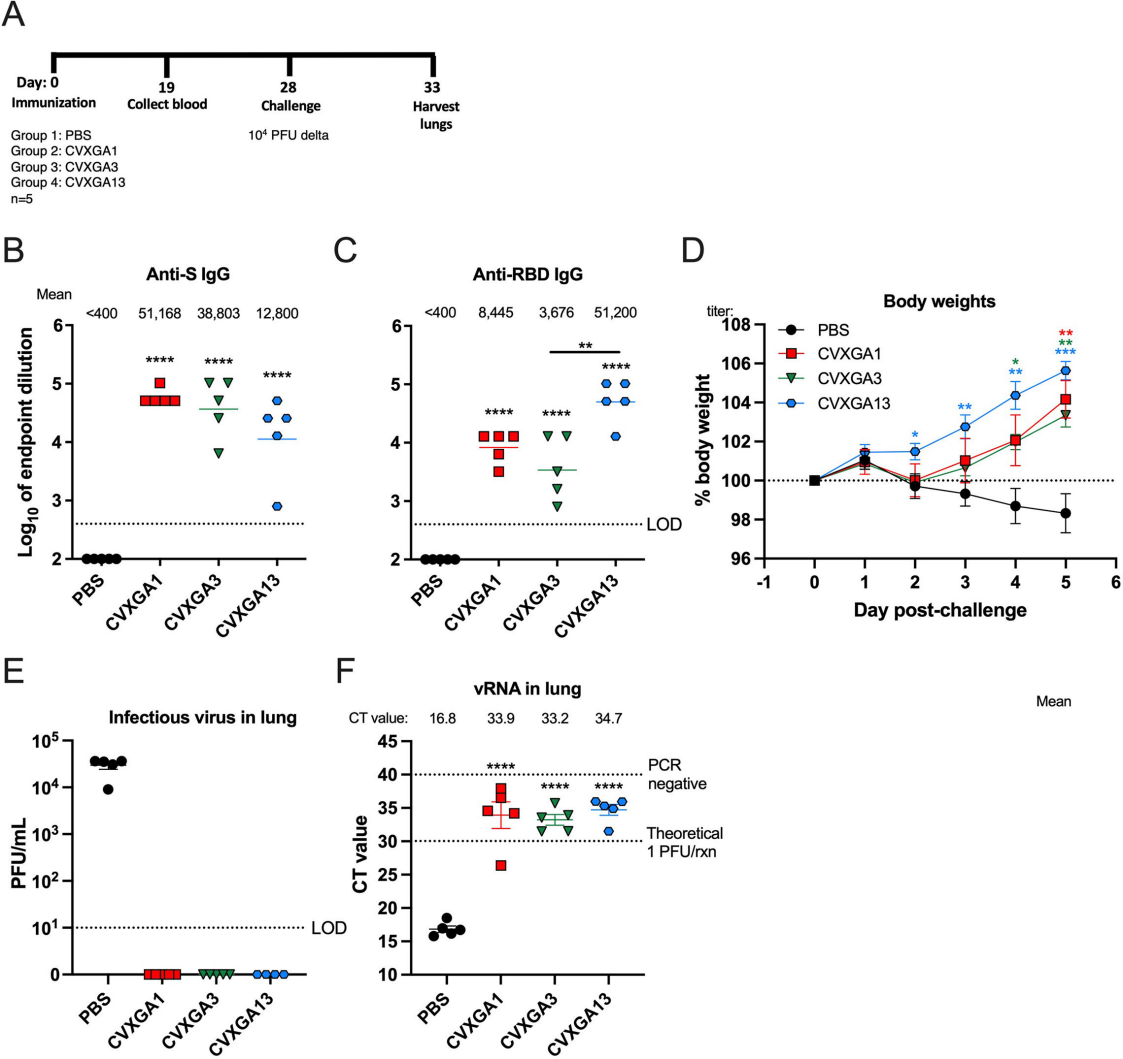
Immunization with CVXGA1 protects hamsters from challenge with SARS-CoV-2 delta variant. (**A**) Schematic of hamster study AE23 immunization. Golden Syrian hamsters (*n* = 5) were intranasally immunized with 100 µL PBS, 3 × 10^5^ PFU CVXGA1, 2 × 10^5^ PFU CVXGA3, or 1.5 × 10^5^ PFU CVXGA13. Blood was collected at 19 dpi. At 28 dpi, the hamsters were challenged with 10^4^ PFU of SARS-CoV-2 delta variant. Following challenge infection, the hamster weights were monitored daily before terminating the study and harvesting lungs at 5 dpc. Anti-SARS-CoV-2 WA1 S (**B**) and RBD (**C**) IgG antibodies were quantified via ELISA at 19 dpi. Antibody titer was calculated as log_10_ of the highest serum dilution at which the OD_450_ was greater than two SDs above the mean OD_450_ of naïve serum. The limit of detection (LOD) is indicated by the dotted line. Error bars represent the SEMs. Comparing each group to mock PBS-immunized hamsters, statistical significance was calculated by nonparametric Kruskal-Wallis multiple comparisons (*P ≤ 0.05, **P < 0.01, and ***P < 0.001). (**D**) Following challenge infection, hamster body weights were monitored daily for 5 days and graphed as percent of day 0 wt. Statistics were calculated for each timepoint by comparing each vaccine group to the PBS group with Mann-Whitney tests (*P ≤ 0.05 and *P* < 0.01). (**E**) Viral load in lung homogenate was quantified via plaque assay in Vero TMPRSS2 cells and graphed as PFU/ml lung homogenate. The LOD is indicated by the dotted line. Error bars represent the SEMs. (**F**) RNA was extracted from lung homogenate, and SARS-CoV-2 delta vRNA was quantified via RT-qPCR. The CT value for each sample is presented, and error bars represent the SEMs. The known viral titer of delta variant was used to generate a standard curve to calculate the CT value equating to 1 PFU/rxn. The LOD, PCR negative, is indicated by a dotted line at CT value = 40, the number of PCR cycles. Comparing each group to mock PBS-immunized hamsters, statistical significance was calculated by nonparametric Kruskal-Wallis multiple comparisons (**P < 0.01).

As of May 2022, delta variant was one of two VOCs circulating in the United States (https://www.who.int/activities/tracking-SARS-CoV-2-variants). Therefore, we assessed the immunogenicity and efficacy of CVXGA1 (WA1 S), CVXGA3 (beta S), and CVXGA13 (delta S) against heterologous delta variant challenge. Serum anti-SARS-CoV-2 WA1 S and S-RBD IgG antibodies were quantified via ELISA at 19 days post-immunization with a single, intranasal dose of PBS or 10^5^ PFU of CVXGA1, CVXGA3, or CVXGA13 (Fig. 5A). CVXGA1, CVXGA3, or CVXGA13 elicited anti-S IgG antibodies with mean titers of over 10,000 (Fig. 5B). The S IgG antibody level from CVXGA13 was lower than CVXGA1 and CVXGA3. However, hamsters immunized with CVXGA13 had the highest level of anti-WA1 RBD antibodies with a mean titer of 50,119 (Fig. 5C).

At 28 dpi, the immunized hamsters were intranasally challenged with 10^4^ PFU SARS-CoV-2 delta variant, and their body weights were monitored for 5 days. Beginning at 2 dpc, hamsters immunized with PBS experienced weight loss that steadily declined until the study was terminated. In contrast, hamsters immunized with CVXGA1, CVXGA3, or CVXGA13 experienced weight gain after 2 dpc. While not statistically significant, hamsters immunized with CVXGA13 had greater weight gain than hamsters immunized with CVXGA1 or CVXGA3, indicating that PIV5 expressing S from SARS-CoV-2 delta variant protected hamsters the best from weight loss following homologous challenge with delta variant (Fig. 5D). To further assess the vaccine efficacy, challenge virus load in lungs at 5 dpc was examined by plaque assay and RT-qPCR. While CVXGA1-, CVXGA3-, or CVXGA13-immunized hamsters did not have detectable infectious challenge virus in their lung homogenates, hamsters immunized with PBS had a mean titer of 3 × 10^4^ PFU/mL lung homogenate (Fig. 5E). Four of five hamsters immunized with CVXGA1 and all hamsters immunized with CVXGA3 or CVXGA13 had CT values indicative of no infectious virus. One CVXGA1-immunized hamster had a CT value of 26.39 indicative of >1 PFU virus/reaction (Fig. 5F). The weight loss and lung viral burden data from this study demonstrated that single, intranasal doses of CVXGA1, CVXGA3, and CVXGA13 protected hamsters from homologous and heterologous challenge. The protective effect offered by CVXGA13 against homologous delta virus is the best among the three CVXGA vaccine candidates based on the highest body weight gain.

### CVXGA1 generates longer-lasting immunity

**Fig 6:**
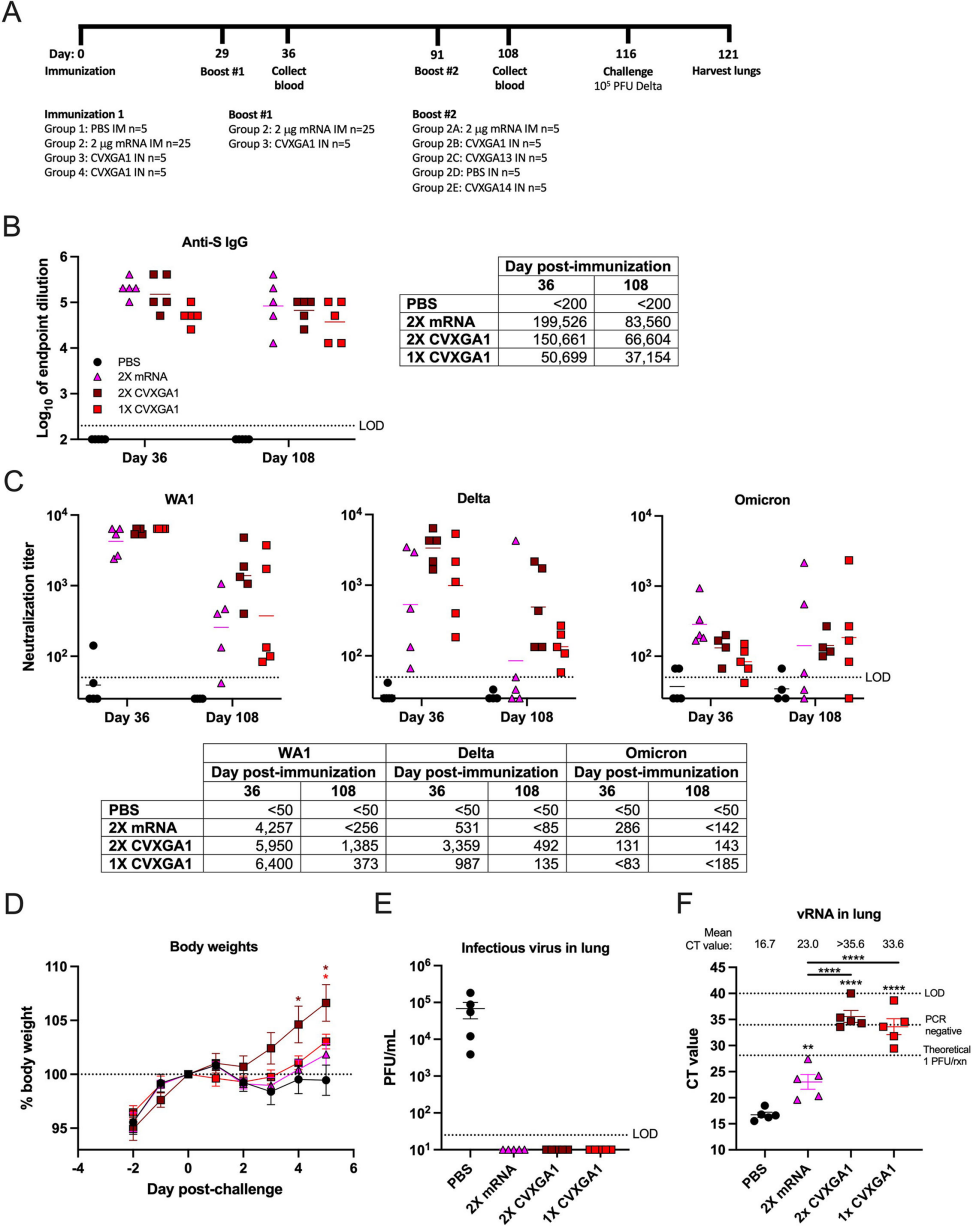
Immunization with a COVID-19 mRNA vaccine, one dose of CVXGA1, or two doses of CVXGA1 protects hamsters against challenge with delta variant. (**A**) Schematic of hamster study AE24 immunization. Golden Syrian hamsters received 100 µL PBS intranasally (*n* = 5, group 1), 2 µg mRNA COVID vaccine (*n* = 25, group 2), or 100 µL 7 × 10^4^ PFU CVXGA1 (*n* = 10, groups 3 and 4). At 29 dpi, hamsters that received the mRNA vaccine were boosted with the mRNA vaccine, and group 3 hamsters received another dose of CVXGA1. At 91 dpi following initial immunization, hamsters who received two doses of mRNA received 2 µg mRNA vaccine (*n* = 5, group 2A), 7 × 10^4^ PFU CVXGA1 (*n* = 5, group 2B), 10^3^ PFU CVXGA13 (*n* = 5, group 2C), PBS IN (*n* = 5, group 2D), or 10^4^ PFU CVXGA14 (*n* = 5, group 2E). Blood was collected at 36 and 108 dpi. At 116 dpi, the hamsters were challenged with 10^5^ PFU SARS-CoV-2 delta variant. Following challenge infection, hamster weights were monitored for 5 days, and the lungs were harvested. (**B**) Anti-SARS-CoV-2 WA1 S IgG antibodies were quantified via ELISA at 36 and 108 days post-immunization. Antibody titer was calculated as log_10_ of the highest serum dilution at which the OD_450_ was greater than two SDs above the mean OD_450_ of naïve serum. The limit of detection (LOD) is indicated by the dotted line. The geometric means are presented in the table and represented as bars on the graph. Comparing each group to mock PBS-immunized hamsters, statistical significance was calculated by nonparametric Kruskal-Wallis multiple comparisons (*P ≤ 0.05 and **P < 0.01). (**C**) Microneutralizing antibody titers against SARS-CoV-2 WA1, delta, or omicron were calculated as log_10_ of the highest serum dilution at which the virus infectivity was reduced by at least 50%. The LOD is indicated by the dotted line. The geometric means are presented in the table and represented as bars on the graph. Comparing each group to mock PBS-immunized hamsters, statistical significance was calculated by nonparametric Kruskal-Wallis multiple comparisons (*P ≤ 0.05 and **P < 0.01). (**D**) Following challenge, hamster weights were monitored daily for 5 days and graphed as percent of day 0 wt. Statistics were calculated for each timepoint by comparing each vaccine group to the PBS group with Mann-Whitney tests (*P ≤ 0.05). (**E**) Viral load in lung homogenate at 5 dpc was quantified via plaque assay in Vero TMPRSS2 cells and graphed as PFU/ml lung homogenate. The LOD is indicated by the dotted line. Error bars represent the SEMs. (**F**) Viral RNA load in lung homogenate was quantified via RT-qPCR. The CT value for each sample is presented, and error bars represent the SEMs. The known viral titer of delta variant was used to generate a standard curve and calculate the CT value equating to 1 PFU/rxn. The CT value generated from RNA extracted from sterile water is denoted by a dotted line labeled PCR negative. The LOD is indicated by a dotted line at CT value = 40, the number of PCR cycles. Error bars represent the SEMs. Statistical significance was calculated by nonparametric analysis of variance with Kruskal-Wallis multiple comparisons (**P < 0.01 and ***P < 0.001).

As of July 2023, 81% of individuals in the United States are fully vaccinated against COVID-19 (4). However, vaccine-induced immunity wanes over time, making vaccines less effective against SARS-CoV-2 variants (5, 6, 9). To assess the longevity of CVXGA1, we compared one (1× CVXGA1) and two (2× CVXGA1) intranasal doses of CVXGA1 to two intramuscular doses of a mRNA COVID-19 vaccine (2× mRNA) in hamsters over time (Fig. 6A). Blood was collected 7 (day 36) and 79 (day 108) days following the second immunization to measure anti-SARS-CoV-2-S antibodies. Levels of anti-S ELISA antibody titers against WA1 in hamsters immunized with two doses of mRNA vaccine were higher than those reported by Meyer et al. (27) but comparable at day 36 after the first dose of mRNA immunization. Hamsters who received 2× mRNA had the highest anti-S ELISA titer, and 2× CVXGA1-immunized hamsters had higher anti-S titers than 1× CVXGA1-immunized hamsters, whose mean anti-WA1 S antibody titer was 50,699 on day 36, but the titers on day 108 are comparable for all three vaccines (Fig. 6B). Neutralizing antibodies against SARS-CoV-2 WA1, delta variant, and omicron variant were determined by microneutralization assay. While 2× mRNA generated a high level of anti-WA1 neutralizing antibody (4,257 at 7 days post-second dose), 2× CVXGA1 generated comparable levels of anti-S ELISA titers but higher levels of anti-WA1 neutralizing antibodies at day 108 (Fig. 6B and C). Consistent with reduced cross-reactivity of antibodies elicited by mRNA immunization, delta-, and omicron-specific neutralizing antibody levels from 2× mRNA were lower, with titers of 531 and 286, respectively. As expected, 1× and 2× CVXGA1 immunization also generated lower neutralization antibody levels against delta and even lower for omicron (Fig. 6C). These data confirmed antigenic drift of the SARS-CoV-2 variants.

The longevity of antibody responses in the immunized hamsters was examined on 79 days after boost (day 108 after initial immunization). SARS-CoV-2 S-specific IgG antibody titers declined during this time to 41.9%, 44.2%, and 73.3% for 2× mRNA, 2× CVXGA1, and 1× CVXGA1, respectively (Fig. 6B). Reduction of neutralizing antibody titers in 2× mRNA-immunized hamsters was substantial with 20%, 60%, and 40% of hamsters having no detectable WA1-, delta-, and omicron-neutralizing antibodies at 79 days post-boost. In contrast, serum from all hamsters immunized with 2× CVXGA1 maintained levels of neutralizing antibodies against WA1, delta variant, and omicron variant better than the 2× mRNA vaccine group (Fig. 6C).

Eighty-seven days after the second immunization, the hamsters were challenged with delta variant, and the hamster weights were monitored for 5 days. Compared to hamsters who were immunized with PBS, hamsters who received two doses of CVXGA1 had significant weight gain following challenge. Hamsters who received two doses of mRNA vaccine and 1× CVXGA1 had body weight gain but not statistically significant from the PBS group (Fig. 6D). Viral burden in the hamster lungs at 5 dpc was tested by plaque assay and RT-qPCR. While PBS-immunized hamsters had mean lung titers of 5.2 × 10^3^ FFU/mL lung homogenate, none of the vaccinated hamsters had a detectable infectious virus in their lung homogenate (Fig. 6E). However, all hamsters immunized with two doses of mRNA vaccine had SARS-CoV-2 vRNA levels indicative of infectious virus with the mean CT value equating to 38 PFU per RT-qPCR reaction (Fig. 6F). This data suggested that CVXGA1 immunized animals had longer-lasting immunity compared to 2× mRNA vaccination.

### Boosting with CVXGA improves protection of hamsters immunized with mRNA COVID-19 vaccine

**Fig 7:**
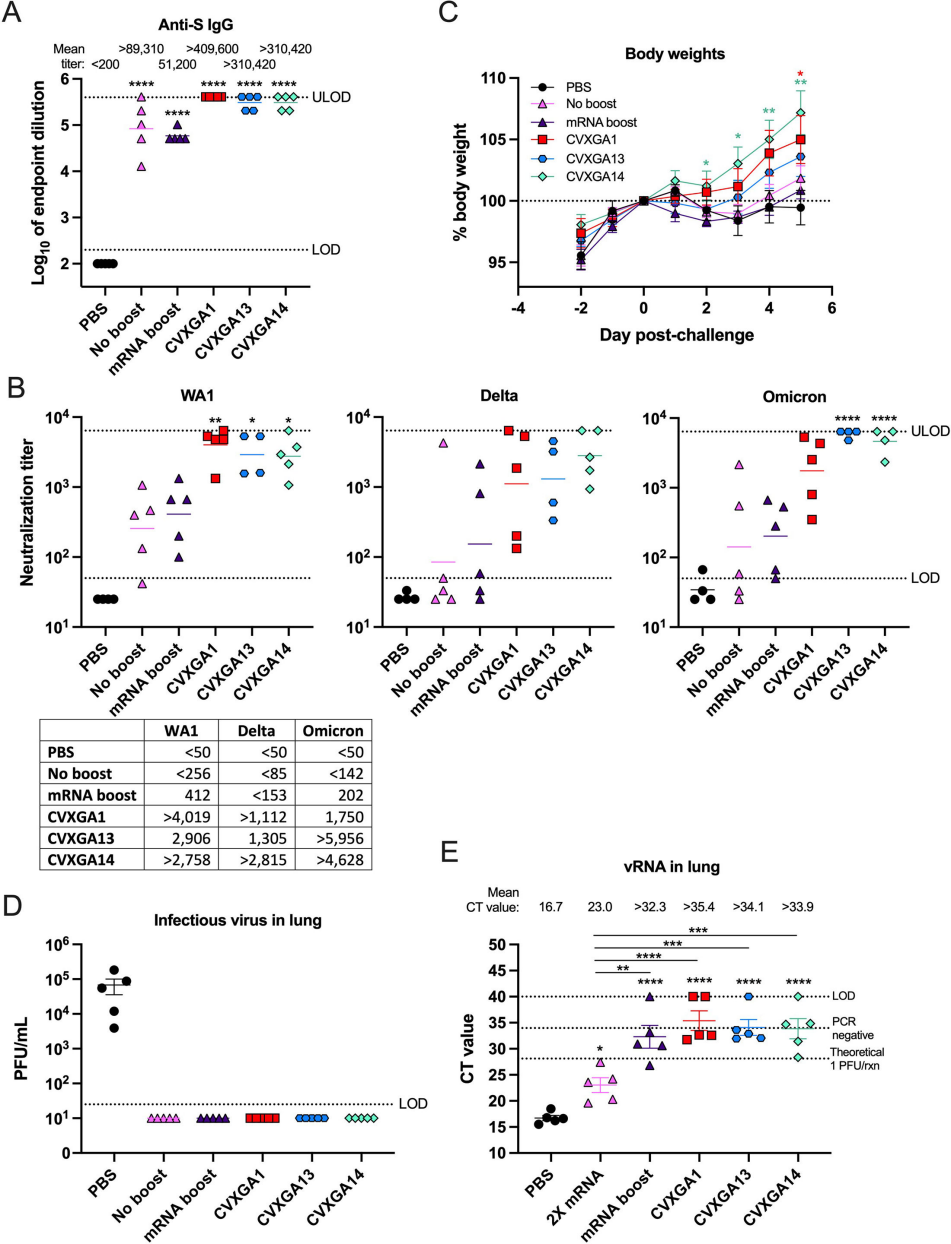
CVXGA1 boosts the humoral immune response and protection of mRNA-immunized hamsters against challenge with delta variant. (**A**) Anti-SARS-CoV-2 S IgG antibodies at 17 days post-boost were quantified via ELISA. Antibody titer was calculated as log_10_ of the highest serum dilution at which the OD_450_ was greater than two SDs above the mean OD_450_ of naïve serum. The limit of detection (LOD) is indicated by the dotted line. Bars represent geometric means. Statistical significance was calculated with nonparametric Kruskal-Wallis multiple comparisons (***P < 0.001 and **P < 0.01). (**B**) Microneutralizing antibody titers against SARS-CoV-2 WA1, delta, or omicron were calculated as log_10_ of the highest serum dilution at which the virus infectivity was reduced by at least 50%. The LOD is indicated by the dotted line. The geometric means are presented in the table and represented as bars on the graph. Statistical significance was calculated between each group and PBS-immunized or mRNA boost groups with nonparametric Kruskal-Wallis multiple comparisons (*P ≤ 0.05 and **P < 0.01). (**C**) Hamster body weight changes over 5 dpc were graphed as a percent of day 0 wt. Statistics were calculated for each timepoint by comparing each vaccine group to the PBS group with Mann-Whitney tests (*P ≤ 0.05). (**D**) Viral load in lung homogenates at 5 dpi was quantified via plaque assay in Vero TMPRSS2 cells and graphed as PFU/ml lung homogenate. The LOD is indicated by the dotted line. Error bars represent the SEMs. (**E**) SARS-CoV-2 delta vRNA load in lung homogenate was quantified via RT-qPCR. The CT value for each sample is presented, and error bars represent the SEMs. The known viral titer of delta variant was used to generate a standard curve and to calculate the CT value equating to 1 PFU/rxn. The CT value generated from RNA extracted from sterile water is denoted by a dotted line labeled PCR negative. The LOD is indicated by a dotted line at CT value = 40, the number of PCR cycles. Error bars represent the SEMs. Statistical significance was calculated between each group and PBS-immunized or mRNA boost groups with nonparametric Kruskal-Wallis multiple comparisons (*P ≤ 0.05 and **P < 0.01).

Due to large populations having already been immunized with COVID-19 vaccines, we investigated the use of CVXGA vaccines (CVXGA1, 13 and 14) as a booster in the hamster model (Fig. 6A, group 2). We used 2× mRNA immunization as a starting point for comparison. Hamsters were first immunized with two doses of mRNA COVID-19 vaccine. Sixty-two days after the second dose of mRNA vaccination, hamsters were boosted with mRNA (total 3× mRNA vaccine doses), CVXGA1, CVXGA3, CVXGA14, or PBS. Hamsters who received intranasal boosts of CVXGA1 (group 2A), CVXGA13 (group 2C), or CVXGA14 (group 2E) had anti-S IgG titers greater than 5 log_10_, while hamsters who received an mRNA vaccine boost (group 2A) or no boost (group 2D) had lower anti-S IgG titers (Fig. 7A). Antibody cross reactivity with WA1, delta variant, and omicron variant measured by neutralization assay showed negligible difference in neutralization titer between sera from hamsters who received a mRNA boost to hamsters who received no boost. However, boosting with mRNA increased detectable levels of neutralizing antibodies against WA1, delta, and omicron from 4, 2, and 3 of 5 animals to 5, 3, and 5 of 5, respectively, indicating that an mRNA boost modestly increased neutralizing antibody responses (Fig. 7B). In contrast, significantly higher levels of neutralizing antibodies against WA1, delta variant, and omicron variant were observed in mRNA-immunized hamsters boosted with CVXGA1, CVXGA13, or CVXGA14 (Fig. 7B).

Twenty-five days following the boost, the hamsters were challenged with delta variant. Hamsters who received intranasal boosts of CVXGA14 or CVXGA1 had the best weight gain compared to hamsters who received PBS, no boost, or an mRNA boost. Interestingly, hamsters who received CVXGA14 (omicron S) experienced higher weight gain (*P* ≤ 0.05 on day 3 and *P* < 0.01 on days 4 and 5) than hamsters who received CVXGA13 (homologous delta S antigen; Fig. 7C). None of the boosted hamsters had detectable infectious virus in their lung homogenate at 5 dpc (Fig. 7D; LOD, 25 PFU/mL). However, high levels of viral RNA were detected in animals with only two doses of mRNA. CVXGA-boosted animals had the least amount of viral RNA, while mRNA-boosted animals had higher levels of viral RNA among all boosted groups (Fig. 7E).

## DISCUSSION

An intranasal PIV5-vectored SARS-CoV-2 vaccine expressing WA1 S (CVXGA1) has been proven efficacious against SARS-CoV-2 WA1 strain in mice and ferrets (14). However, WA1 is no longer circulating globally, and it is important for next-generation COVID-19 vaccines to induce immunity against VOCs. Although subvariants of omicron VOC were circulating in the US at the time of this study, omicron VOC causes minimal disease and does not replicate well in hamsters (21). Instead of doing a challenge study with omicron, we measured omicron-specific cross-neutralizing antibodies (Fig. 6C) and demonstrated that one intranasal immunization of CVXGA1 protects hamsters against homologous WA1 and heterologous alpha and delta virus challenges (Fig. 4 to 6). Additionally, CVXGA2, expressing both S and N antigens of SARS-CoV-2 WA, provided increased protection following homologous and heterologous challenge (Fig. 4), and its mechanism of protection might be attributed to N-protein medicated cellular immune responses.

Approximately 81% of the US population has been vaccinated with a COVID-19 vaccine (4), but the approved COVID-19 vaccines are less efficacious against variants due to waning immunity and antigenic drift (7, 8). We wanted to evaluate if CVXGA1 was able to boost pre-existing anti-COVID-19 immunity to provide protection against variants. In this study, we demonstrated that boosting 2× mRNA-immunized hamsters with an intranasal dose of CVXGA1 resulted in an approximately 4.6-fold increase of anti-WA1 S antibody levels (Fig. 7A) and a significant increase of WA1-, delta-, and omicron-neutralizing antibody titers (>15.7-, >13.1-, and >12.3-fold, respectively; Fig. 7B) better than mRNA boost, indicating that intranasal CVXGA1 immunization can enhance VOC-specific immunity in previously vaccinated individuals.

Increased immune escape has led to the recommendation of updating COVID-19 vaccines to currently circulating VOCs (7, 8, 28). In addition to assessing CVXGA1 efficacy against SARS-CoV-2 VOCs, we generated PIV5-vectored vaccines expressing the spike protein from beta, gamma, delta, or omicron and evaluated their immunogenicity and protection against homologous and heterologous SARS-CoV-2 virus challenge. Indeed, vaccination with PIV5-vectored delta spike protein (CVXGA13) resulted in the most weight gain following challenge with delta variant (Fig. 5D). PIV5-vectored vaccines expressing WA1, beta, and gamma spike protein (CVXGA1, CVXGA3, and CVXGA5, respectively) induced similar levels of anti-WA1 S IgG antibodies (Fig. 3A) and provided similar protection against body weight loss following challenge with WA1 or alpha variant (Fig. 4A and B). These data indicate antigenic distinction and variation in pathogenicity among WA1, alpha, beta, gamma, and delta variants. Boosting 2× mRNA-immunized hamsters with PIV5 expressing delta (CVXGA13) or omicron S (CVXGA14) substantially increased neutralizing antibody levels against VOCs WA1, delta variant, and omicron (Fig. 7B), indicating that boosting with a PIV5-vectored vaccine can serve as a booster vaccine against VOCs.

Vaccine-induced local immunity in the upper airways is an important mechanism of protection for controlling virus spread to the lungs and for reducing person-to-person transmission of respiratory viruses. We acknowledge the limitation of our hamster study that did not address mucosal and cellular immunity, as the studies focused on the protection of various vaccine candidates and boosting the ability of the intranasal vaccine. We have addressed the protection of CVXGA1 in a nonhuman primate (NHP) model and showed CVXGA1 offered significant protection against challenge infection in the upper and lower respiratory tracts (accompanying publication). We have attempted to evaluate SARS-CoV-2-S-specific cellular immune response by ELISpots using hamster splenocytes and mucosal immunity by ELISA for hamster IgA in hamster tracheal washes. However, limited golden Syrian hamster reagents are available, and the acquired reagents did not generate reliable data. CVXGA1-induced cellular and mucosal immunity have been demonstrated in an NHP model, and the data can be found in the accompanying paper.

Pre-existing vector-specific immunity is a general concern for viral-vectored vaccines. A previous study showed that approximately 30% of the human population is seropositive for PIV5, potentially due to exposure to dogs vaccinated with the kennel cough vaccine that contains live PIV5. The same study also showed that immunization with a PIV5-vectored vaccine can overcome pre-existing anti-PIV5 immunity in dogs (29). In this study, a single immunization with CVXGA1 protects hamsters against WA1 and VOCs alpha and delta, and 2× CVXGA1 generates longer-lasting immunity (Fig. 6C), higher body weight gain (Fig. 6D), and lower viral RNA levels after challenge (Fig. 6F), indicating CVXGA1’s boosting effect. The effect of PIV5 vector immunity on CVXGA1 and PIV5-vectored RSV vaccines in humans is currently being evaluated in respective clinical trials.

Several intranasal COVID-19 vaccines, including adenovirus- (30), Newcastle disease virus- (31), parainfluenza virus 3 (PIV3)- (32), and influenza- (33) vectored vaccines, have been studied clinically or preclinically, and several have shown improved immunogenicity when administered intranasally compared to intramuscularly (30, 34). The approval of intranasal COVID-19 vaccines by India for adenovirus-vectored vaccine iNCOVACC (35) and China for adenovirus-vectored vaccine Convidecia (36) and influenza virus vectored vaccine dNS1-RBD (33) provides promise for the intranasal vaccine platform.

In summary, CVXGA1, an intranasal vaccine currently being evaluated in human clinical trials in the US (15, 16), protects against challenge with homologous SARS-CoV-2 virus strain and heterologous VOCs as a single dose in naïve and mRNA-immunized hamsters. Our data suggest that CVXGA1, and other PIV5-vectored COVID-19 vaccines, can serve as an effective heterologous booster to offer long-lasting, cross-reactive immunity to individuals who have received COVID-19 mRNA vaccines.

## Data Availability

All data needed to evaluate the conclusions in the paper are present in the paper and/or the supplemental material. SARS-CoV-2 can be provided by S.M.T. pending scientific review and a completed material transfer agreement. Requests for the SARS-CoV-2 should be submitted to the University of Georgia Research Foundation. CVXGA1 can be provided by B.H. pending scientific review and a completed material transfer agreement. Requests for the CVXGA1 should be submitted to the University of Georgia Research Foundation. Additional data related to this paper may be requested from the authors.

## SUPPLEMENTAL MATERIAL

Figure S1 jvi.01989-24-s0001.pdf Standard curve for RT-qPCR.

ASM does not own the copyrights to Supplemental Material that may be linked to, or accessed through, an article. The authors have granted ASM a non-exclusive, world-wide license to publish the Supplemental Material files. Please contact the corresponding author directly for reuse.
