# Supplementary material for: Efficacy of parainfluenza virus 5 (PIV5)-vectored intranasal COVID-19 vaccine as a single dose primer and booster against SARS-CoV-2 variants
Source: J Virol. 2025 Mar 21;99(4):e01989-24. doi: 10.1128/jvi.01989-24 (PMC11998504; doi:10.1128/jvi.01989-24)

Supplemental figure

Supplemental figure 1. RT-qPCR standard curves. RNA was extracted from SARS-CoV-2 WA1 (A), alpha variant (B), and delta variant (C) viral stocks of known titer. The RNA was serially diluted and vRNA was quantified via RT-qPCR. To generate a standard curve, the Ct value was plotted on the y-axis and the PFU per reaction (rxn) was plotted on the x-axis. Dotted lines indicate the Ct value which corresponds to 1 PFU/rxn.

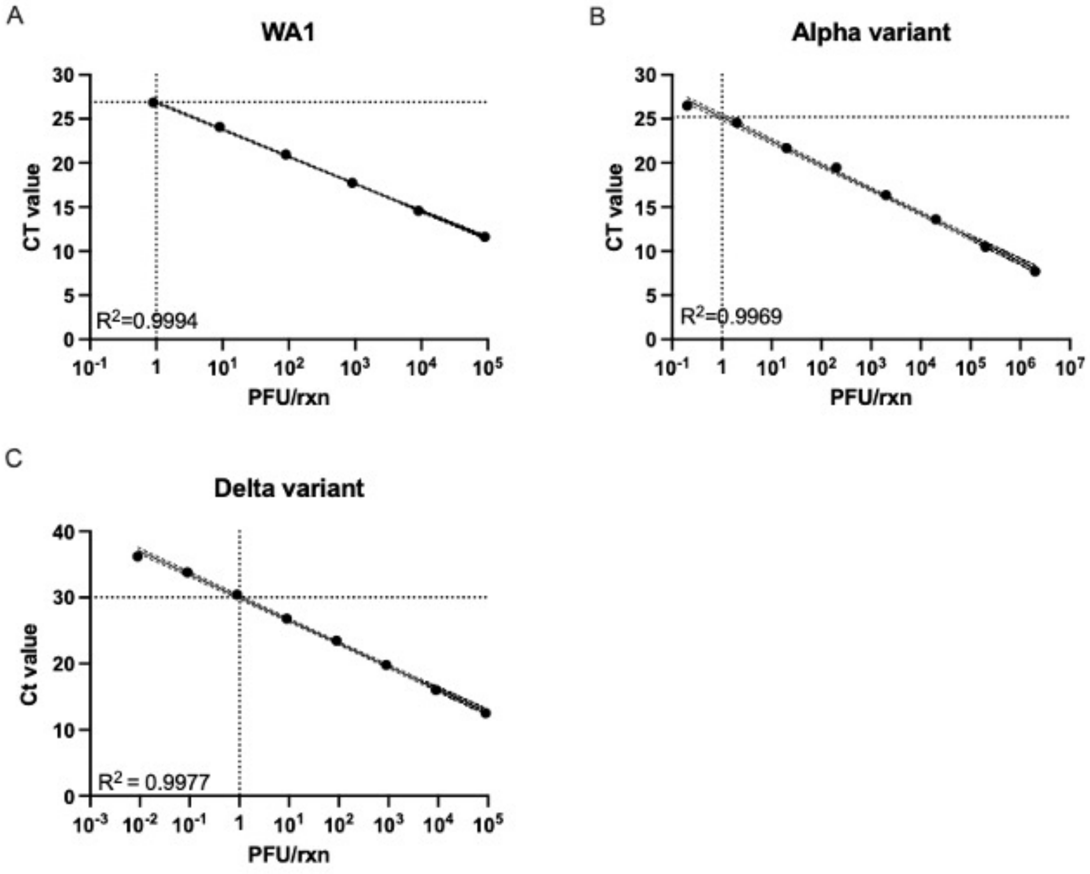

Supplement: Figure S1 — Standard curve for RT-qPCR. [file jvi.01989-24-s0001.pdf]
